# Editorial on Special Issues “Aerogels” and “Aerogels 2018”

**DOI:** 10.3390/gels6030019

**Published:** 2020-06-29

**Authors:** Françoise Quignard, Nathalie Tanchoux

**Affiliations:** ICGM, Univ Montpellier, CNRS, ENSCM, 34296 Montpellier, France; quignard@enscm.fr

Aerogels can be defined as ultralight materials with a 3D porous structure, similar to their parent wet gels, where the solvent has been replaced by a gas without a collapse of the gel structure, thanks to the drying process used (supercritical CO_2_ drying, freeze drying, etc.). These amazing materials usually present advantages in terms of surface area, diffusion properties, thermal conductivity, refractive index, and dielectric constant, which confer those exciting properties exploitable in many fields. The scope of applications for aerogels is broad and goes from insulation (thermal, acoustic) to storage and absorbing media, sensors, regenerative medicine, pharmaceuticals, and catalysis. The most publicized application to date is the use of silica-based aerogels by NASA as the insulating material in the Mars rover robot, used to gather information on the surface of Mars. The earliest aerogels synthesized were made of silica, but soon, many inorganic-based aerogels appeared, along with aerogels based on synthetic polymers (polyurea-based aerogels, for example), on biopolymers—often called bioaerogels—or aerogels based on hybrid organic–inorganic composite structures.

The field of research concerning aerogels is then huge and exciting and has grown tremendously in the past 20 years, corroborated by the number of occurrences found through the Web of Science search engine, which retrieved 137 references on aerogels and bioaerogels in 1999 vs. 1420 references in 2019. This book includes 16 articles and reviews published in the two Special Issues “Aerogels” and “Aerogels 2018”, published in *Gels* in 2016 and 2018. These articles and reviews cover a wide range of subjects, going from the more fundamental aspects to the applicative possibilities offered by the development of aerogels. Together, these papers provide a nice overview of the fields covered by research on aerogels, along with a perspective on the challenges and opportunities for future developments in the field.

Silica aerogels—the first type of aerogels synthesized—and their derivatives constitute a wide field of research. Understanding/modifying the structures and properties of silica-based aerogels is especially important to improve their mechanical properties to widen their application fields. To address this issue, M. Schwan et al. describe in their article [1] the synthesis of a new composite material with silica aerogel reinforced by aramid honeycomb materials. This association leads to composite materials keeping the low thermal conductivity necessary for their use as insulators, while tremendously increasing their mechanical strength. In the same spirit, L. Duraes et al. studied in their article [2] the influence of structure directive additives (glycerol and polyethylene glycol) on the properties of silica aerogel-like monoliths, prepared from methyltrimethoxysilane precursor. The use of such additives allows tuning of the structural properties of these aerogels, rendering them suitable for thermal insulation applications. In their article, S.C. Joshi et al. [3] present the synthesis of flat silica aerogels composites, using porcine-gelatin binders as well as different additives (fumes silica, carbon nanotubes) along with their mechanical—compression and flexure—properties. M. Sachithanadam et al. present the acoustic properties of various silica aerogels and silica aerogels—sodium dodecyl sulfate composites—showing the remarkable performances of silica aerogel composites for acoustic insulation applications [4]. Another type of application, the use of silica aerogels as supports for gold nanoparticles, is described in the article presented by I. Lázár et al. [5]. This work investigates the different reaction parameters needed to prevent the aggregation and preserve the size of gold nanoparticles. The contribution by T. Woignier et al. [6] presents the sintering of silica aerogels into dense silica porous glass. This new way of synthesis allows elimination of the larger pores without changing the internal silica structure, thus leading to porous glasses with different physical properties, such as acoustical properties, thermal conductivity or permeability. In another review, presented by T. Woignier et al. [7], the relationships between the porous structure of silica aerogels and their mechanical properties are described, from a more fundamental point of view. It shows that aerogels are elastic and brittle materials, and porosity is the main parameter, which controls the mechanical properties. More precisely, porosity, pore size distribution and OH content are necessary parameters to describe completely the aerogels’ brittle behavior.

Besides silica aerogels, other types of inorganic, organic or hybrid aerogels also attracted the attention of researchers as promising materials. For example, C. Macias et al. reported the synthesis of carbon aerogels prepared by sol-gel polycondensation of melamine–resorcinol–formaldehyde (MRF) mixtures with incorporation of diatomite and carbon black additives [8]. The resulting aerogel composites displayed interesting properties, leading to the preparation of flexible monolithic carbon aerogel electrodes with a good performance in the electrochemical removal of ions from solution. L. Ratke et al. present a more fundamental study on the effect of gelation kinetics on the size of resorcinol–formaldehyde aerogels particles [9]. This experimental and theoretical study shows a clear dependence of gelation time on the sample size under mild shear conditions, which allowed the development of a theoretical model that agrees with experimental findings. In another contribution, P. Paraskevopoulou et al. present the synthesis of millimetric polyurea aerogel beads with a narrow size distribution [10]. The new synthetic method developed in this work is simple, cost-efficient and suitable for large-scale production of PUA aerogel beads. Another study on aerogels based on synthetic polymers is presented by H. M. Duong et al. [11]. The authors present here a simple, practical and scalable approach to fabricating polyethylene terephthalate (PET) aerogels from recycled PET fibers, polyvinyl alcohol (PVA) and glutaraldehyde (GA) crosslinkers, for thermal and acoustic insulation applications. In another study, D. A. Shiraldi et al. [12] showed that polyvinyl alcohol aerogels synthesized with binary nanofillers (clay and graphite) exhibit low thermal and electrical conductivities, even with high loading of graphite. D. A. Shiraldi also described in a further article a more fundamental study on the relationship between the rheological properties of gels and the mechanical properties of their corresponding aerogels, in the case of highly porous clay/poly(vinyl alcohol) composite aerogels [13].

Aerogels based on biopolymers exhibit also many promising properties and constitute an exciting development field. In their article, R. Subrahmanyam et al. describe, from a fundamental point of view [14], the interaction between the biopolymer matrix (calcium alginate, here) and the solvent during solvent exchange and prior to supercritical CO_2_ drying. This shows the importance of the choice of solvent, its concentration, and the kinetics of exchange in biopolymer aerogel production. In the review presented by P. Gurikov et al. [15], the recent advances in non-conventional gelation methods of alginate are critically discussed, especially cryotropic gelation, non-solvent-induced phase separation and carbon dioxide-induced gelation.

Finally, C. Erkey et al. describe in their review the experimental and theoretical studies on the kinetics of supercritical drying found in the literature [16], as this step is the most important one in aerogel formation.
